# Molecular Biomarkers of Malignant Transformation in Head and Neck Dysplasia

**DOI:** 10.3390/cancers14225581

**Published:** 2022-11-15

**Authors:** Kushi Ranganath, Allen L. Feng, Ramon A. Franco, Mark A. Varvares, William C. Faquin, Matthew R. Naunheim, Srinivas Vinod Saladi

**Affiliations:** 1 Department of Otolaryngology-Head & Neck Surgery, Harvard Medical School, Boston, MA 02115, USA; 2 Department of Otolaryngology-Head & Neck Surgery, Massachusetts Eye & Ear, Boston, MA 02114, USA; 3 Broad Institute of MIT and Harvard, Boston, MA 02114, USA

**Keywords:** head and neck cancer, head and neck squamous cell carcinoma, dysplasia, biomarker, molecular marker, tumor microenvironment

## Abstract

**Simple Summary:**

Head and neck cancer patients face significant morbidity and mortality. Early detection and diagnosis of disease followed by timely intervention is necessary for improving clinical management for these patients. There remains a need to be able to identify whether an early pre-cancerous lesion at the dysplasia stage will progress to invasive cancer. Biomarkers are biological molecules found in blood or tissue that are measurable at early stages of disease and can be applied to predict the progression of such lesions. The aim of this review is to comprehensively present the available evidence on the most frequently altered tumor molecular biomarkers present in head and neck dysplasia as well as their potential clinical applications.

**Abstract:**

Head and neck squamous cell carcinoma (HNSCC) and its treatments are associated with substantial morbidity, often resulting in cosmetic deformity and loss of physiologic functions including speech and swallowing. Despite advancements in treatment, 5-year survival rates for mucosal malignancies remain below 70%. Effective prevention of HNSCC demands an understanding of the molecular pathways of carcinogenesis. Specifically, defining features of pre-cancerous dysplastic lesions that indicate a better or worse prognosis is necessary to help identify patients who are likely to develop a carcinoma and allow a more aggressive approach to management. There remains a need for identification of biomarkers that can provide both early prognostic and predictive value in clinical decision-making by serving as both therapeutic targets as well as predictors of therapy response. Here, we comprehensively review the most frequently altered molecular biomarkers of malignant transformation in head and neck dysplasia. These markers are involved in a wide range of cellular processes in head and neck carcinogenesis, including extracellular matrix degradation, cell motility and invasion, cell–cell adhesion, solute transport, immortalization, metabolism, the cell cycle and apoptosis, transcription, and cell signaling.

## 1. Introduction

Head and neck squamous cell carcinoma (HNSCC) is the fifth most common cancer, accounting for approximately 5% of all malignancies worldwide [1,2]. Head and neck cancer encompasses cutaneous malignancies of the face and scalp as well as tumors arising from mucosal surfaces of the upper aerodigestive tract, namely the oral cavity, nasopharynx, oropharynx, hypopharynx, and larynx [3]. When diagnosis is delayed, which is the case in over half of HNSCC patients, HNSCC carries a poor prognosis and is associated with substantial mortality [4]. Despite advancements in treatment, 5-year survival rates for mucosal malignancies remain below 70% [5]. HNSCC and its treatments are also associated with substantial morbidity, often resulting in cosmetic deformity and loss of physiologic functions including speech and swallowing [3].

Effective prevention of HNSCC demands an understanding of the molecular pathways of carcinogenesis. The most important risk factors for HNSCC include tobacco and alcohol consumption and Human Papillomavirus (HPV) infection [6]. Prolonged exposure to these carcinogens drives genetic and epigenetic alterations that result in progression from dysplasia to carcinoma in situ to invasive carcinoma [7]. The current clinical practice for assessing at-risk lesions within the head and neck is histopathologic examination for dysplasia and carcinoma [8]. The current grading of dysplasia has several limitations, notably that the stepwise nature of dysplasia classification does not reflect the more continuous evolution of this disease process [9,10]. Furthermore, dysplasia refers to a heterogeneous group of cytologic and architectural changes, with not all cases progressing to invasive carcinoma or following the classic ordered histologic progression model [11,12]. At this time, histologic characteristics cannot reliably determine whether a dysplastic lesion will progress. Defining features of dysplastic lesions that indicate a better or worse prognosis is necessary to refine clinical management [12]. Such prognostic biomarkers may help identify patients who are likely to develop a carcinoma and allow a more aggressive approach to management.

**Figure 1:**
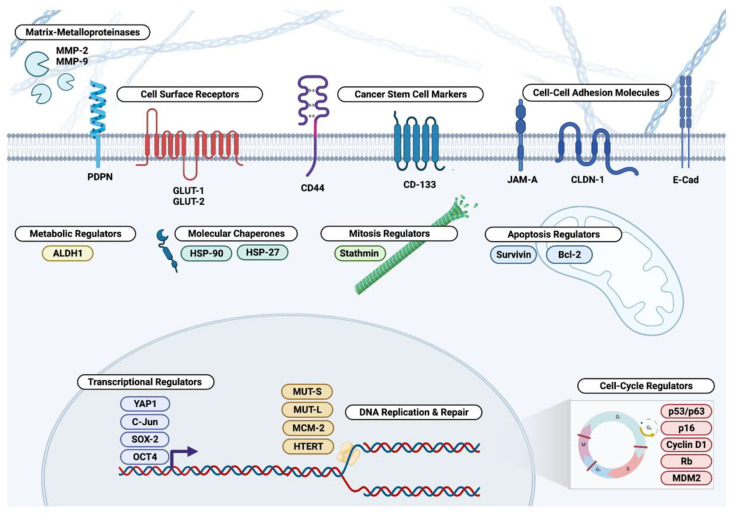
Molecular biomarkers in head and neck dysplasia. Biomarkers include matrix metalloproteinases, cell surface receptors, cancer stem cell markers, cell adhesion molecules, transcriptional regulators, DNA replication and repair proteins, and cell cycle regulators. Created with BioRender.

There remains a need for the identification and validation of novel biomarkers that can predict the progression of pre-malignant lesions [13]. However, biological markers in HNSCC are not as well-studied as they are in other tumor types, such as breast or pancreatic cancer. To that end, we summarize the genetic progression model of HNSCC followed by a comprehensive review of molecular biomarkers of malignant transformation in head and neck dysplasia with an emphasis on oral cavity and laryngeal dysplasia (Figure 1). 

## 2. Genetic Mechanisms Underlying Progression of Dysplasia to Carcinoma

In the classic histologic progression model of HNSCC, lesions proceed from normal mucosa to epithelial hyperplasia to low- or high-grade dysplasia to carcinoma in situ (CIS) to invasive carcinoma. Of note, not all dysplasia progress in a histologically recognizable stepwise fashion through this model. The risk of developing invasive carcinoma increases with progression of histologic grade. 

**Figure 2:**
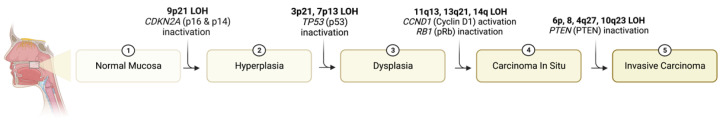
Genetic progression model of head and neck squamous cell carcinoma. Accumulation of loss of heterozygosity or amplification at various genetic loci occurs as lesions proceed from normal mucosa to hyperplasia to dysplasia to carcinoma in situ to invasive carcinoma. Created with BioRender.

A subset of the genetic events that drive the progression from one stage to the next is outlined here. Accumulation of loss of heterozygosity (LOH) at multiple genetic loci leads to histopathologic progression [14]. LOH at 9p21 occurs early as normal mucosa becomes proliferative and develops the histologic appearance of hyperplasia. This results in inactivation of the associated tumor suppressor genes cyclin-dependent kinase inhibitor 2A (CDKN2A), which encodes the CDK inhibitor p16INK4A, and ARF, which encodes the p53 stabilizer p14. LOH at the 3p and 17p chromosome regions occurs at the subsequent transition from hyperplasia to dysplasia. The most common loci that are lost include 3p14, 3p21, and 17p13, and their associated tumor suppressor genes fragile histidine triad gene (FIHT), Ras association domain family 1 isoform A (RASSF1A), and TP53, respectively. Amplification of 11q13 and resultant overexpression of cyclin D1 as well as LOH of 13q21 and resultant inactivation of the retinoblastoma gene occurs at the transition from dysplasia to CIS. LOH at 6p, 8, 4q27, and 10q23 leads to the inactivation of tumor suppressors including phosphatase and tensin homolog (PTEN) and marks the transition from CIS to invasive carcinoma [14,15,16,17,18] (Figure 2). Notably, this genetic progression occurs within the context of a field effect, where tissue adjacent to lesions share genetic alterations and undergo histopathologic changes that may result in synchronous or metachronous tumors [14].

## 3. Extracellular and Cell Surface Biomarkers

### 3.1. Matrix Metalloproteinases 

Matrix metalloproteinases (MMP) are extracellular proteinases which degrade extracellular matrix and basement membrane. They play roles in regulating tissue homeostasis, wound healing, immunity, inflammation, and angiogenesis. Alterations in MMP activity are implicated in chronic inflammatory diseases and cancer [19]. MMP-2 and MMP-9 are type IV collagenases [20] that dissolve collagen IV, the main structural component of basement membrane [21]. Breach of this barrier between epithelium and underlying mesenchyme is a key event in the progression from dysplasia to invasive carcinoma [22]. Studies have reported overexpression of MMP-2 and MMP-9 in oral epithelial dysplasia (OED) [23], with MMP-9 expression correlating significantly with OED grade in patient samples [24,25]. Levels of MMP-1 and MMP-9 mRNA are specifically elevated in OED cases that progress to cancer compared to those which do not [26]. MMPs also regulate the early tumor microenvironment, with findings of high MMP expression in both tumor and stromal cells which function as “initiators” and “promoters” of carcinogenesis, respectively [27]. Stromal cells include fibroblasts, macrophages, and vascular endothelial cells that secrete MMPs into the extracellular space [28]. The extracellular MMP inducer (EMMPRIN) stimulates MMP synthesis in stromal fibroblasts at early stages of tumorigenesis, and EMMPRIN expression also correlates with the grade of OED patient samples [29]. MMP-9 expression and tumor vascularity progressively increase from normal mucosa to OED, indicating an additional pro-angiogenic role of MMPs in early tumor formation [24]. Ultimately, MMP overexpression mediates early tumor invasion, epithelial to mesenchymal transition (EMT), and angiogenesis. 

### 3.2. Podoplanin 

Podoplanin (PDPN) is a mucin-like transmembrane glycoprotein that plays roles in the development of the lungs, lymphatic system, and heart as well as the biology of immune cells and platelet activation [30]. PDPN overexpression has been reported in various human squamous cell carcinomas, particularly oral cavity, larynx, skin, cervical, and lung cancers [31]. PDPN is expressed in 90% of oral squamous cell carcinomas (OSCC) [32] and 37% of oral leukoplakias (OL) [33]. PDPN expression has been found to correlate with the degree of OED in patient samples and predicts a higher rate of progression to oral cancer [33,34,35,36]. In tumor cells, PDPN enables migration and invasion by modulating the actin cytoskeleton. PDPN induces tumor invasion by mediating single-cell migration following EMT or collective cell migration in the absence of EMT [37]. It is present at the invasive peripheral edge of approximately 80% of human squamous cell carcinomas [38,39]. Acquisition of PDPN expression at the tumor front may therefore drive invasion into surrounding tissue during tumor initiation. 

### 3.3. Claudin, JAM-A, and E-Cadherin 

Cell–cell adhesion is critical to maintain a barrier against tumor invasion. Epithelial tight junctions are comprised of claudins (CLDN-1), occludins, and junctional adhesion molecules (JAM-A) [40]. Overexpression of CLDN-1 and JAM-A has been reported in OED and OSCC patient samples and correlates with histologic grade. The mechanism by which upregulation of these proteins leads to tumorigenesis is not fully elucidated. However, findings of CLDN-1 and JAM-A delocalization from the cell membrane to cytoplasm during malignant transformation suggests their possible role in cell signaling [40]. Studies in other tumor types posit that CLDN-1 promotes MMP activity leading to cell migration, and JAM-A induces EMT [40,41,42]. Adherens junctions are another key component of epithelial tissue architecture and are comprised of E-cadherins, calcium-dependent adhesion proteins [43]. In addition to stabilizing cell–cell interactions [44], E-cadherin mediates signaling pathways involved in cellular proliferation, differentiation, and apoptosis [43,45]. E-cadherin expression has been found to decrease with increasing grades of dysplasia in OL patient samples [43,46,47]. Loss of the “invasion suppressor” E-cadherin is therefore a critical step in EMT and tumor invasion in oral carcinogenesis [43,48].

### 3.4. CD44 and CD133

CD44 is a cell surface transmembrane glycoprotein involved in cell migration, adhesion, leukocyte and lymphocyte activation, myelopoiesis and lymphopoiesis, and angiogenesis [49]. Alternative splicing of the CD44 gene results in standard (CD44s) and variant (e.g., CD44v3, CD44v6) isoforms which interact with ligands including hyaluronate, osteopontin, collagens, and metalloproteinases [49,50]. CD44s is expressed in most human tissues, whereas variant isoforms are more limited in distribution and often expressed in response to oncogenic signals [49,51]. CD44 is a well-known cancer stem cell (CSC) marker [51]. While some studies report CD44s overexpression in OED [52] and dysplastic oral lichen planus (OLP) [53], others found reduced CD44v6 expression in OED patient samples [54]. Decreased CD44 expression was also observed in non-tumor epithelium adjacent to oral and laryngeal cancers, suggesting an early role for CD44 in carcinogenesis [55,56,57]. Taken together, there is no consistent pattern of CD44 expression in HNSCC, with observations of both CD44 gain and loss [49,58]. Nonetheless, dysregulation of CD44 expression alters the early proliferative status and adhesive properties of tumor cells [49].

CD133, also known as Prominin-1, is a cell surface transmembrane glycoprotein and the most frequently used CSC marker [59]. The physiologic function of CD133 is unclear, although it may be involved in membrane organization, autophagy, signal transduction (IL-8, mTOR, PI3K, MAPK), cellular scaffolding, and glucose metabolism [60]. In CSCs, it plays critical roles in self-renewal, cell growth, differentiation, and metabolism [59,60]. Increased CD133 expression has been reported in OED and OSCC relative to normal mucosa in patient samples [61,62]. CD133 overexpression is also significantly associated with malignant progression to carcinoma in 80% of OLP cases [63] and 59% of OL cases [64]. Furthermore, CD133 delocalization from the cell membrane to the nucleus or cytoplasm also correlates with malignant transformation [65].

### 3.5. Glucose Transporters

Glucose transporters (GLUT) facilitate glucose and fructose transport across the cell membrane, thereby regulating cellular energy metabolism [66,67]. Rapidly proliferating cancer cells have a high ATP and glucose requirement, resulting in the upregulation of GLUT and glycolytic enzymes to meet this demand [66]. Glycolysis is especially advantageous to promote tumor survival under hypoxic conditions and when energy supply is limited, which is often the case in carcinogenesis [67,68]. In addition to functioning as an insulin-dependent glucose transporter, GLUT-4 is an activator of glucose-independent signaling pathways that promote migration and invasion [69]. Studies have reported overexpression of GLUT-1 and GLUT-4 in OED relative to OSCC [68,70], and increases in GLUT-1 expression also correlate with degree of dysplasia in patient samples [67]. Increased expression in dysplastic over carcinomatous lesions suggests GLUT upregulation occurs early in oral tumorigenesis [68,71].

## 4. Cytosolic Biomarkers

### 4.1. Aldehyde Dehydrogenases

Metabolic dysregulation is a consistent feature of tumor cells [72]. Aldehyde dehydrogenase (ALDH) is a cytosolic enzyme involved in the oxidation of aldehydes to carboxylic acids, essential for reduction of oxidative stress [73]. Certain isoforms of ALDH (e.g., ALDH1A1, ALDH1A2, ALDH1A3, ALDH8A1) oxidize retinol to retinoic acid (RA), resulting in RA cell signaling that induces the “stemness” of CSCs [74]. ALDH1 is a well-known CSC marker in HNSCC [75]. Its expression correlates with the number of cells undergoing EMT and therefore reflects the invasive potential of a tumor [76]. Levels of ALDH1 expression have been found to increase significantly from dysplasia to OSCC in association with histologic grade in patient samples [73,76]. ALDH1 expression is associated with a 4.17-fold increase in the risk of malignant transformation of OL, with 48.1% of ALDH1-positive OL clinical cases progressing to invasive carcinoma [64].

### 4.2. Molecular Chaperones

Heat shock proteins (HSP) are molecular chaperones that regulate protein folding, refolding of misfolded proteins, protein repair and degradation, and intracellular protein transport [77,78]. Cellular stressors such as low nutrient supply, oxidative stress, hypoxia, injury, and apoptotic signals can induce HSP synthesis [77]. Of the five major families of HSPs, HSP70, and HSP90 are the most frequently identified to play a role in OSCC. HSP70 and HSP90 expression have been found to increase with increasing grades of OED and OSCC in patient samples [79,80,81].

### 4.3. Mitosis and Apoptosis Regulators

The microtubular cytoskeleton is critical to the integrity of mitosis. Stathmin is a cytosolic phosphoprotein that is involved in the regulation of the microtubule cytoskeleton. Depending on its phosphorylation state, stathmin regulates both entry into and exit from mitosis via modulation of mitotic spindle assembly [82]. Increased stathmin expression has been reported in various tumor types [83,84] including nasopharyngeal carcinoma and OSCC [85,86]. Stathmin expression increases with increasing grades of OED in patient samples, suggesting an early role in tumorigenesis [87,88].

Apoptosis is essential to the physiologic turnover of human tissue [89]. Regulators of apoptosis can be divided into the Bcl-2 family and inhibitors of apoptosis (IAP) family [90]. Bcl-2 anti-apoptotic proteins inhibit mitochondrial outer membrane permeabilization, thereby limiting the release of cytochrome c and resultant caspase activation [91,92]. Increased Bcl-2 expression is an early event in carcinogenesis and is observed in 30–60% of OED [89,93]. Detection of Bcl-2 has also been used to discriminate OL from OLP with high specificity in clinical samples, suggesting its potential role as a marker of malignant transformation [94]. IAPs regulate apoptosis downstream of Bcl-2 by functioning as endogenous inhibitors of caspases [92]. Survivin is one member of the IAP family that is expressed in 40–70% of OL patient cases [95,96]. Survivin positivity has also been reported in 94% of dysplasia patient cases that transformed to OSCC [97].

## 5. Nuclear Biomarkers

### 5.1. Cell Cycle Regulators

The p53 tumor suppressor is encoded by the TP53 gene on chromosome 17 [98]. Mutations in p53 are detectable in 50% of human cancers [99]. The p53 family of transcription factors induces the expression of genes needed for cell cycle arrest, DNA repair, apoptosis, cellular senescence, and metabolism. This classically occurs in response to DNA damage, oxidative stress, and hypoxia [100]. P53 acts at the G1/S cell cycle checkpoint where it activates the p21-Rb-E2F pathway. In G1, hyperphosphorylation of retinoblastoma protein (Rb) by cyclin-dependent kinases (CDK) releases E2F transcription factors from the Rb-E2F repressor complex and drives cells into S phase [101]. At this point, cell cycle arrest by p53 is primarily mediated by activation of the CDK inhibitors p21, p15, and p16 [92,98,102]. P53 target genes such as 14-3-3σ also mediate arrest at the subsequent G2/M transition [103]. MDM2 is a negative regulator of p53 that functions as a E3 ubiquitin ligase that marks p53 for proteasomal degradation [104].

**Figure 3:**
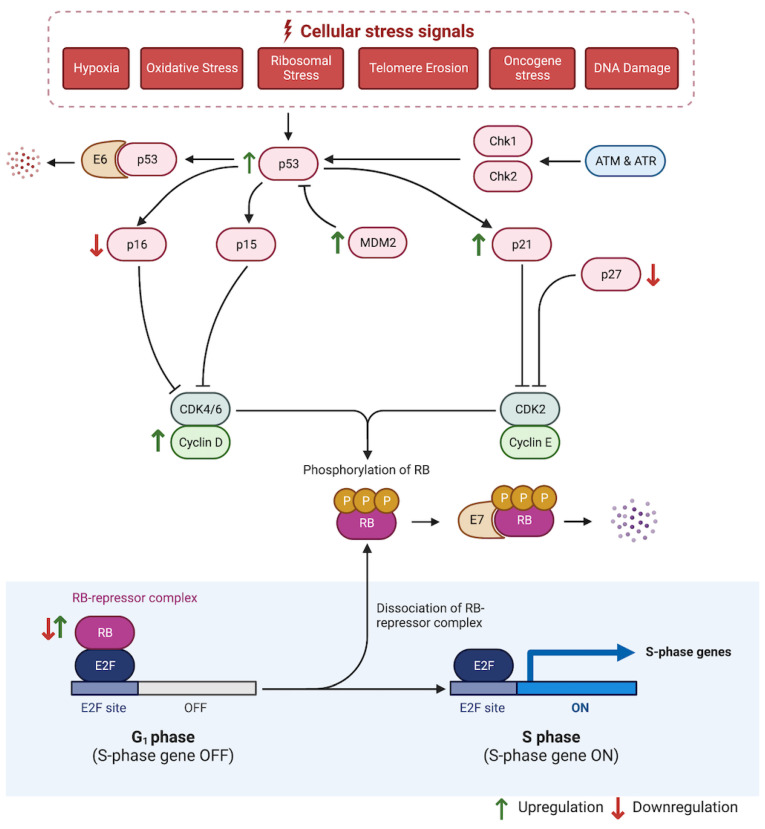
p53 tumor suppressor pathway. p53 acts on the G1/S checkpoint of the cell cycle to mediate cell cycle arrest via activation of CDK inhibitors. Arrows represent alterations in head and neck dysplasia. Adapted from “G1/S Checkpoint” and “The p53-Mediated Response,” by BioRender.com (2022) (https://app.biorender.com/biorender-templates (accessed on 10 August 2022)).

Levels of p53 expression increase in association with histologic grade of OED [105]. In fact, p53 expression is present in 80% of oral epithelial hyperplasia [106] and increases significantly from OL to OSCC in patient samples [81]. P53 expression is also associated with progression to malignancy in laryngeal dysplasia [107]. Expression of the p53 homologs, p63 and p73, is increased in OED relative to normal mucosa [108,109,110]. Overexpression of other cell cycle regulators including cyclin D1, Rb, and MDM2 have been reported in OL relative to normal mucosa [111]. Of the G1/S regulators, loss of p16 is the earliest event, and subsequent loss of pRb, gain of p53, and gain of cyclin D1 are associated with malignant progression [112,113] (Figure 3).

### 5.2. Transcriptional Regulators

**Figure 4:**
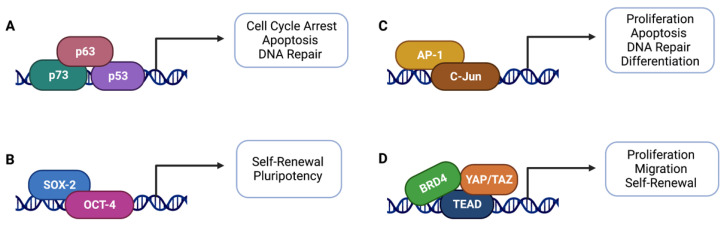
Transcriptional regulators in head and neck dysplasia: (**A**) p53, p63, and p73; (**B**) SOX-2 and OCT-4; (**C**) AP-1 and C-Jun; (**D**) BRD-4, YAP, TAZ, and TEAD. Created with BioRender.

C-Jun is a transcriptional activator of the activator protein 1 (AP-1) transcription factor family [114] that mediates proliferation, apoptosis, DNA repair, and differentiation [114,115]. C-Jun expression levels have been found to correlate with the severity of oral dysplasia [116,117]. Observations of increased C-Jun expression in dysplasia cases which progressed to carcinoma suggest its early role in carcinogenesis [118]. SOX-2 and OCT-4 are transcription factors that induce the self-renewal and pluripotency properties of embryonic stem cells [119]. Levels of SOX-2 and OCT-4 expression also increase with progression from dysplasia to OSCC in patient samples [34,120]. SOX-2 and OCT-4 overexpression may be necessary to prevent apoptosis [121,122] and regulate EMT in tumor cells, respectively [122] (Figure 4).

### 5.3. DNA Replication and Repair Regulators

Dysregulation of DNA replication and repair processes contributes to the genomic instability of tumor cells. Upregulation of telomerase, an RNA-dependent DNA polymerase that maintains telomere length, has been observed in many tumor types. The catalytic protein component of telomerase is known as human telomerase reverse transcriptase (hTERT). Findings of increased hTERT expression with progression from normal mucosa to OSCC suggests it may promote immortalization of tumor cells [123]. Minichromosome maintenance complex component 2 (MCM2) is a member of the MCM family of proteins which function as replication initiation factors and promote DNA elongation [124]. MCM2 expression is also known to increase progressively in association with the severity of OED in clinical samples [125].

DNA mismatch repair genes, notably MutSα and MutLα, play a similarly important role in preserving genomic integrity and are reduced in oral dysplasia and carcinoma compared to normal mucosa [126]. Ataxia telangiectasia mutated (ATM) and RAD-3 related (ATR) proteins and their downstream checkpoint kinases Chk-2 and Chk-1, respectively, play a similarly critical role in regulating the response to DNA damage. ATM-Chk2 and ATR-Chk1 signaling pathways activate cell cycle checkpoint pathways which induce cell cycle arrest and DNA repair [127]. ATM expression has been found to increase with progression from OL to OSCC and is independently associated with a significantly increased risk of malignant transformation. However, levels of Chk-2 expression do not correlate with the degree of OED, suggesting that it may be regulated differently [128]. Significantly reduced levels of expression of phosphorylated ATR and Chk-1 have been observed in patients with OED progressing to OSCC [129]. Taken together, there are limited studies suggesting an inconsistent pattern of both upregulation and downregulation of DNA repair molecules. Nonetheless, it appears that dysregulation of DNA mismatch repair processes occurs early in oral carcinogenesis.

### 5.4. Hippo Pathway

**Figure 5:**
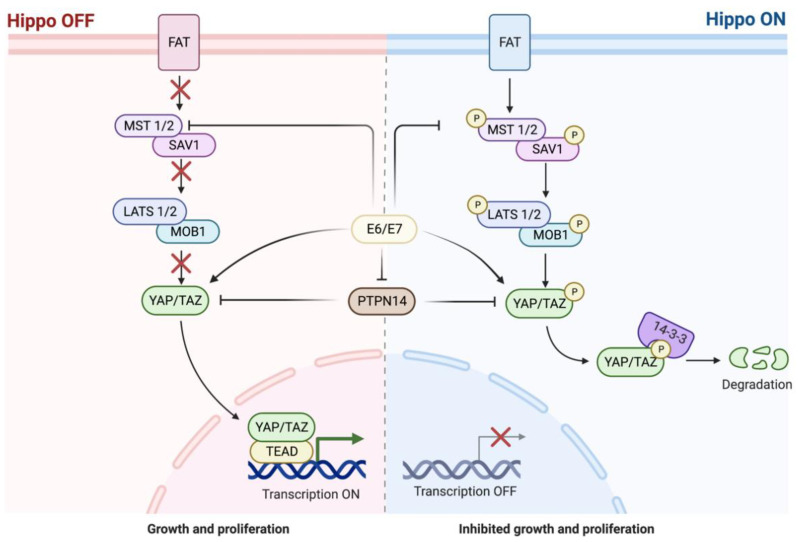
Hippo pathway with Human Papilloma Virus E6/E7 Oncoproteins. Adapted from “The Hippo Tumor Suppressor Pathway” and “Hippo Pathway in Mammals”, by BioRender.com (2022) (https://app.biorender.com/biorender-templates (accessed on 10 August 2022)).

The transcriptional co-regulators Yes-associated protein (YAP) and WW domain-containing transcription regulator protein 1 (WWTR1), hereafter referred to as TAZ, are major effectors of the Hippo pathway that interact with the transcriptional enhanced associate domain (TEAD) family of transcription factors. When the Hippo pathway is off, YAP/TAZ are unphosphorylated and bind TEAD to induce transcriptional programs for cellular proliferation, survival, and migration. When the Hippo pathway is on, inhibitory phosphorylation of YAP/TAZ results in their retention and degradation in the cytoplasm [130] (Figure 5). Somatic alterations of the Hippo pathway have been reported in nearly 50% of HNSCC. FAT1 is an upstream YAP1 inhibitor that is mutated in nearly 30% of HNSCC, and FAT1 deletion has been proposed to contribute to a hybrid EMT state, tumor stemness, and metastasis [131]. We have also recently shown that YAP1 maintains an active chromatin state in HNSCC and promotes tumorigenesis through cooperation with BRD4 [132]. In HPV-related HNSCC, HPV oncoproteins E6 and E7 also activate YAP nuclear localization, promote YAP, TAZ, and TEAD as well as their transcriptional targets, and inhibit upstream YAP1 inhibitors (e.g., PTPN14, MST 1/2) [133,134]. YAP has been found to be elevated in correlation with histologic grade of OED in clinical samples and has been recognized as a potent driver of tumorigenesis in a mouse model [109,135].

## 6. Conclusions and Clinical Applications 

In the dysplasia-to-carcinoma sequence of HNSCC, the presence of dysplasia significantly increases the risk cancer. However, not all dysplastic lesions are the same. Histologic grade cannot reflect all precancerous changes present in a lesion and therefore is not a robust predictor of malignant transformation [136]. For this reason, identification of molecular biomarkers that can discriminate lesions at increased risk of progression is paramount. Most studies have focused on identifying biomarkers in cancer, with comparatively fewer studies of biomarkers altered at earlier stages. We have provided a comprehensive review of the most frequently altered tumor markers present in head and neck dysplasia. These markers are involved in a wide range of cellular processes in head and neck carcinogenesis, including extracellular matrix degradation, cell motility and invasion, cell-cell adhesion, solute transport, immortalization, metabolism, the cell cycle and apoptosis, transcription, and signaling pathways [137] (Table 1). 

The findings of this review suggest that molecules involved in early head and neck tumorigenesis are detectable and measurable, and they may have applications as diagnostic biomarkers for the early detection of cancer. There is not yet enough evidence to support an ordered model of molecular progression in head and neck cancer. Future studies performing molecular phenotyping of cancer cells at each stage of progression are necessary. Molecular biomarkers of high-risk precancerous lesions that correlate with transformation to SCC can provide prognostic value in clinical decision-making. The use of these molecular markers in conjunction with other early clinical and histologic indicators may reliably predict disease progression [138]. The validation of prognostic models which significantly predict malignant transformation and recurrence based on specific histologic biomarkers (e.g., basal cell hyperplasia, loss of epithelial cohesion) has been successful and is ongoing [139]. Ultimately, validation of a prognostic scoring system incorporating molecular markers, clinical risk factors, and histologic grading will provide the greatest clinical utility.

As the field advances toward personalized cancer therapy, there has been an increasing emphasis on identifying predictive biomarkers that can serve as both therapeutic targets as well as predictors of therapy response. For example, we have recently shown that in a subpopulation of HNSCC patients with FAT1 mutations, YAP1, and bromodomain-containing protein 4 (BRD4) can be therapeutically targeted with BET bromodomain inhibitors [132]. Similarly, studies have shown that deficiencies in DNA mismatch repair predict a positive response to immune checkpoint inhibition as well as platinum-based chemotherapies in HNSCC [140,141]. This is also true of cell cycle regulators, as amplification of cyclin D1 predicts cisplatin resistance, TP53 mutations predict susceptibility to G2-M cell cycle inhibitors [142], and Bcl-2 overexpression predicts radiotherapy failure with 71% accuracy [139]. Various therapeutic biomarkers, ranging from viral oncoproteins (e.g., HPV, EBV) and receptor tyrosine kinases (e.g., EGFR, PIK3CA) to immune checkpoint markers (e.g., PD-L1, PD-L2) and tumor suppressor proteins (e.g., TP53) have been identified as therapeutic targets in HNSCC [142]. This work has expanded treatment options in HNSCC to include surgery, radio- and chemotherapy, immune checkpoint inhibition, and molecular targeted therapy [143]. However, there are comparatively fewer studies testing the vulnerability of premalignant cells to these therapies [144]. Therapeutic interventions for HNSCC may not be equally efficacious in pre-cancerous head and neck lesions. For example, EGFR targeted therapy, which has been approved for HNSCC treatment, was not found to have comparable efficacy when used in patients with oral premalignant lesions [145,146]. Identifying molecular based strategies specifically for precancerous lesions is paramount for prevention of HNSCC, and the biomarkers reviewed here provide a future direction as potential therapeutic targets. 

Despite their promising clinical applications, as few as 0.1% of biomarkers are successfully translated into routine clinical practice due to several limitations in current studies: (1) variation in methodology for measurement of marker expression; (2) heterogeneity of cancer samples and study populations; (3) small sample sizes; (4) limited sensitivity, specificity, and predictive value of markers; (5) absence of rigorous clinical validation of markers [147,148]. Future standardized studies addressing these limitations are needed before biomarker tests can be recommended for clinical use. Ultimately, elucidating the cellular, molecular, and genetic events in head and neck carcinogenesis and associated tumor markers is critical to improving the management of HNSCC.

## Figures and Tables

**Table 1:** Histopathologic markers of malignant transformation in head and neck dysplasia.

Biomarker Category	Member (s)	Physiologic Function	Role in Tumorigenesis
Extracellular and Cell-Surface			
Extracellular Degradation	MMP-2, MMP-9	Type IV Collagenase	Tumor invasion
Cell Motility	Podoplanin	Transmembrane Glycoprotein	Tumor invasion
Cell-Cell Adhesion	Claudin	Tight Junction Protein	Migration and MMP induction
	JAM-A	Tight Junction Protein	EMT
	E-Cadherin	Adherens Junction Protein	EMT
Solute Transport	GLUT-1, GLUT-4	Glucose Transporter	Cellular energy supply
Cancer Stem Cell Markers	CD44	Transmembrane Glycoprotein	Proliferation Tumor invasion
	CD133	Transmembrane Glycoprotein	Membrane organization Signal transduction Glucose metabolism MMP induction
Cytosolic Markers			
Metabolic Regulators	ALDH-1	Phase-I Oxidase	Induction of cell “stemness” EMT
Molecular Chaperones	HSP70, HSP90	Heat Shock Proteins	Regulation of protein folding, transport, and repair
Mitosis Regulators	Stathmin	Cytoskeleton Phosphoprotein	Regulation of entry into and exit from mitosis
Apoptosis Regulators	Bcl-2	Inhibitor of Apoptosis	Inhibition of mitochondrial outer membrane permeabilization and apoptosis
	Survivin	Inhibitor of Apoptosis	Inhibition of caspases
Nuclear Markers			
Cell Cycle Regulators	p53 p63 p16 Cyclin D1 pRb MDM2	Tumor Suppressor Tumor Suppressor Tumor Suppressor Inhibitor of pRb Tumor Suppressor E3 Ubiquitin Ligase	Uncontrolled proliferation
Transcriptional Regulators	C-Jun	AP-1 Transcription Factor	Cell proliferation
	SOX-2/OCT-4	Reprogramming Transcription Factors	Self-renewal, de-differentiation
	YAP/TAZ	Hippo Pathway Mediator	Cell proliferation and migration
DNA Replication and Repair Regulators	hTERT	Telomerase Protein	Maintains telomere length Immortalization of tumor cells
	MCM2	Replication Initiation Factor	Immortalization of tumor cells
	MutSα MutLα	DNA Mismatch Repair Protein	Reduced genomic integrity

## Data Availability

Not applicable.
